# Oxidative Stress: Molecular Mechanisms, Diseases, and Therapeutic Targets

**DOI:** 10.1002/mco2.70600

**Published:** 2026-01-29

**Authors:** Yi Qin, Chen Qian, Wenhao Li, Qihan Wang, Qifeng Sheng, Zheqing Chen, Wei Zhang, Wenming Li, Gaoran Ge, Zhanjun Yan, Dechun Geng

**Affiliations:** ^1^ Department of Orthopedics The First Affiliated Hospital of Soochow University, Soochow University Suzhou China; ^2^ Department of Orthopedics Suzhou Ninth Hospital Affiliated to Soochow University Suzhou China

**Keywords:** oxidative damage, oxidative stress, reactive oxygen species, redox signaling, therapeutic targets

## Abstract

Although the physiological level of reactive oxygen species (ROS) is crucial for governing life processes through redox signaling, the excessive accumulation of ROS can contribute to biomolecular damage and pathological state, namely, oxidative stress. This review systematically summarizes the molecular mechanisms underlying the dynamic equilibrium of cellular redox state, including the intracellular sources of ROS and the multilayered antioxidant defense network. When ROS production exceeds the regulatory limits of the antioxidant system, excessive ROS will act on a series of molecular targets and participate in the pathogenesis of diseases. Therapeutic targeting of the redox balance is regarded as an effective strategy for treating oxidative stress‐related diseases, such as supplementation of direct antioxidants and enhancement of endogenous antioxidant defense network. Nevertheless, clinical trials that attempt to delay the onset or progression of such diseases are mostly negative. This review discusses the challenges encountered in the clinical application of antioxidant therapy and highlights the opportunities brought by novel technologies such as intelligent drug delivery system and personalized medicine. By adopting these new technologies, it is expected to overcome the limitations of traditional antioxidant therapy.

## Graphical abstract

Oxidative stress occurs when the reactive oxygen species (ROS) production overwhelms the ROS scavenging, which lead to genomic instability, epigenetic regulation, proteostasis imbalance, and lipid peroxidation, subsequently causing the occurrence and development of numerous diseases.

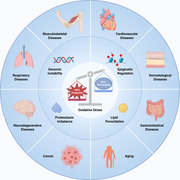

## 1 Introduction

Oxidative stress indicates a state of imbalance between oxidation and antioxidation in the organism. The cellular oxidation and reduction system, also known as the redox system, is an indispensable component of various cellular processes. The acquisition of energy is closely related to the redox system, among which energy supply comes from the oxidative respiration within cells [1]. In this process, a large quantity of reactive oxygen species (ROS) with oxidizing capabilities can be generated. ROS are partially reduced derivatives of molecular oxygen and mainly divided into two groups: highly unstable oxygen free radicals such as superoxide anion radicals (O_2_•^−^) and chemically stable nonradical oxidants such as hydrogen peroxide (H_2_O_2_) [2]. ROS can also be scavenged by the antioxidant system, including the antioxidant enzymes and the non‐enzymatic antioxidants [3, 4]. Under physiological conditions, there exists a delicate balance between the production and elimination of ROS in the body that keeps ROS at low levels [5]. Physiological levels of ROS are essential for various biological processes, such as cell proliferation, differentiation, and apoptosis. However, excessive ROS generation can contribute to molecular damage and pathological state, denoted as oxidative stress [6].

Oxidative stress has been clarified to be related to the occurrence and progression of numerous diseases, including musculoskeletal diseases, cardiovascular diseases, respiratory diseases, dermatological diseases, neurodegenerative diseases, gastrointestinal diseases, cancer, and the aging process [7, 8, 9, 10, 11, 12, 13, 14, 15, 16, 17, 18, 19, 20, 21, 22]. The accumulation of ROS can directly damage biomolecules within cells, resulting in cellular dysfunction or even death [23]. In addition to direct oxidative damage, the dysregulation of redox system leads to aberrant redox modifications and the activation of downstream signaling pathways, including genomic instability, epigenetic regulation, proteostasis imbalance, and lipid peroxidation [24, 25, 26]. Supplementation of direct antioxidants is a classic antioxidant strategy. However, it is extremely difficult to precisely deliver antioxidants to the sites damaged by oxidative stress at the appropriate stage of disease progression [27, 28]. Moreover, the reaction rate of most antioxidants with ROS is much slower than that of antioxidant enzymes, and their actual antioxidant efficiency in vivo may be lower than expected [29, 30]. Enhancement of endogenous antioxidant defense network, such as the nuclear factor‐E2‐related factor 2 (NRF2) pathway and antioxidant enzymes, seems more promising, but it also needs to confront the problem of off‐target. Given the dual role of oxidative stress in both physiological and pathological processes, overly simplified broad‐spectrum antioxidant interventions may fail to achieve the desired therapeutic effect, which also explains the disappointing results in clinical trials [31]. Therefore, the main research goals in the future lie in the intelligent drug delivery system and personalized medicine.

**FIGURE 1:**
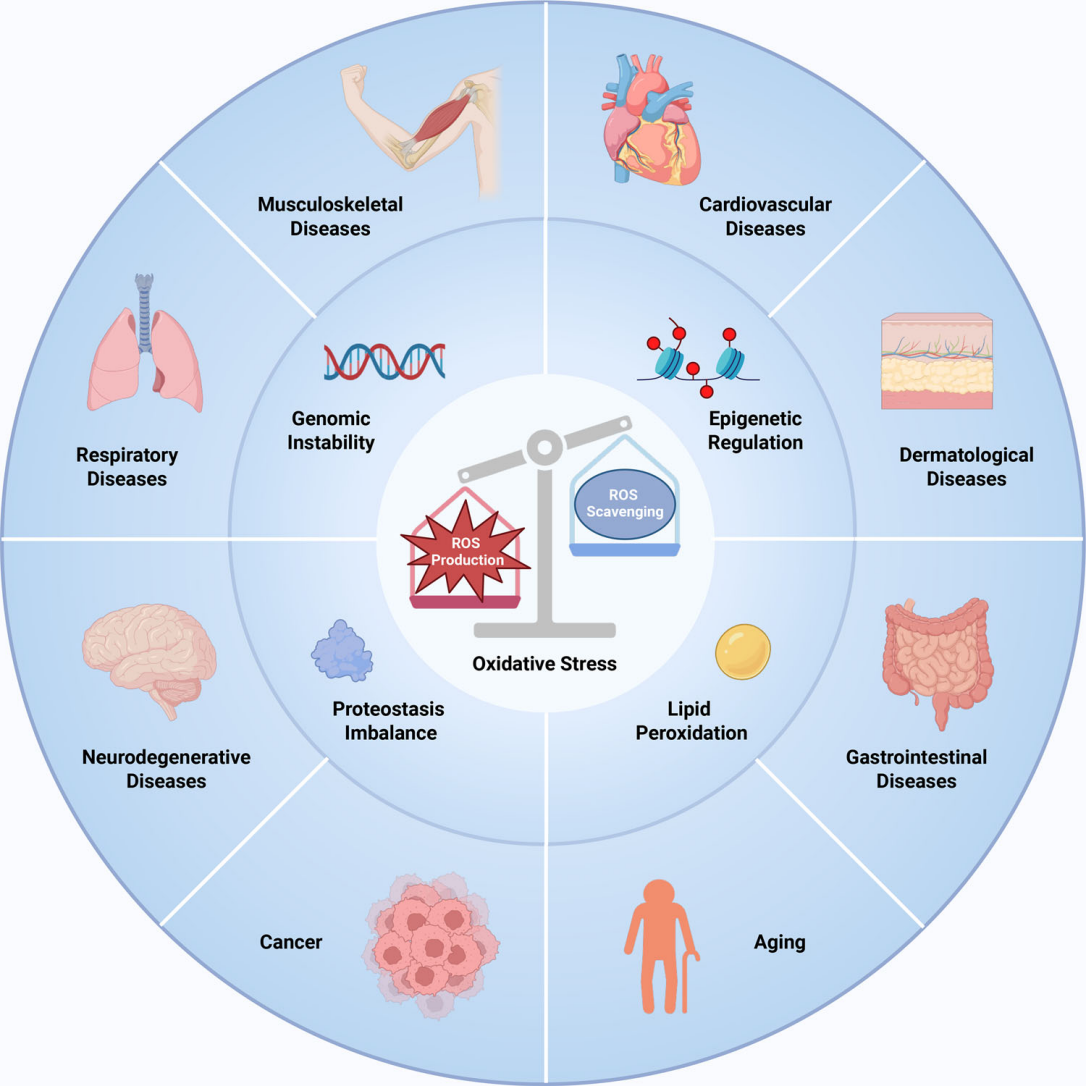
Imbalance of cellular redox homeostasis. Oxidative stress occurs when the reactive oxygen species (ROS) production overwhelms the ROS scavenging, which lead to genomic instability, epigenetic regulation, proteostasis imbalance, and lipid peroxidation. On this basis, oxidative stress is involved in numerous diseases, including musculoskeletal diseases, cardiovascular diseases, respiratory diseases, dermatological diseases, neurodegenerative diseases, gastrointestinal diseases, cancer, and the aging process.

This review will focus on elaborating the molecular mechanisms underlying the dynamic equilibrium of cellular redox state, including the intracellular sources of ROS and the multilayered antioxidant defense network, and clarify the molecular targets of oxidative stress and their roles in the pathogenesis of disease. Furthermore, this review points out the limitations of traditional antioxidant treatments, such as supplementation of direct antioxidants and enhancement of endogenous antioxidant defense network, and highlights the opportunities brought by new technologies, aiming to provide directions for future research in this field (Figure 1).

## 2 The Dynamic Equilibrium of Cellular Redox State

**FIGURE 2:**
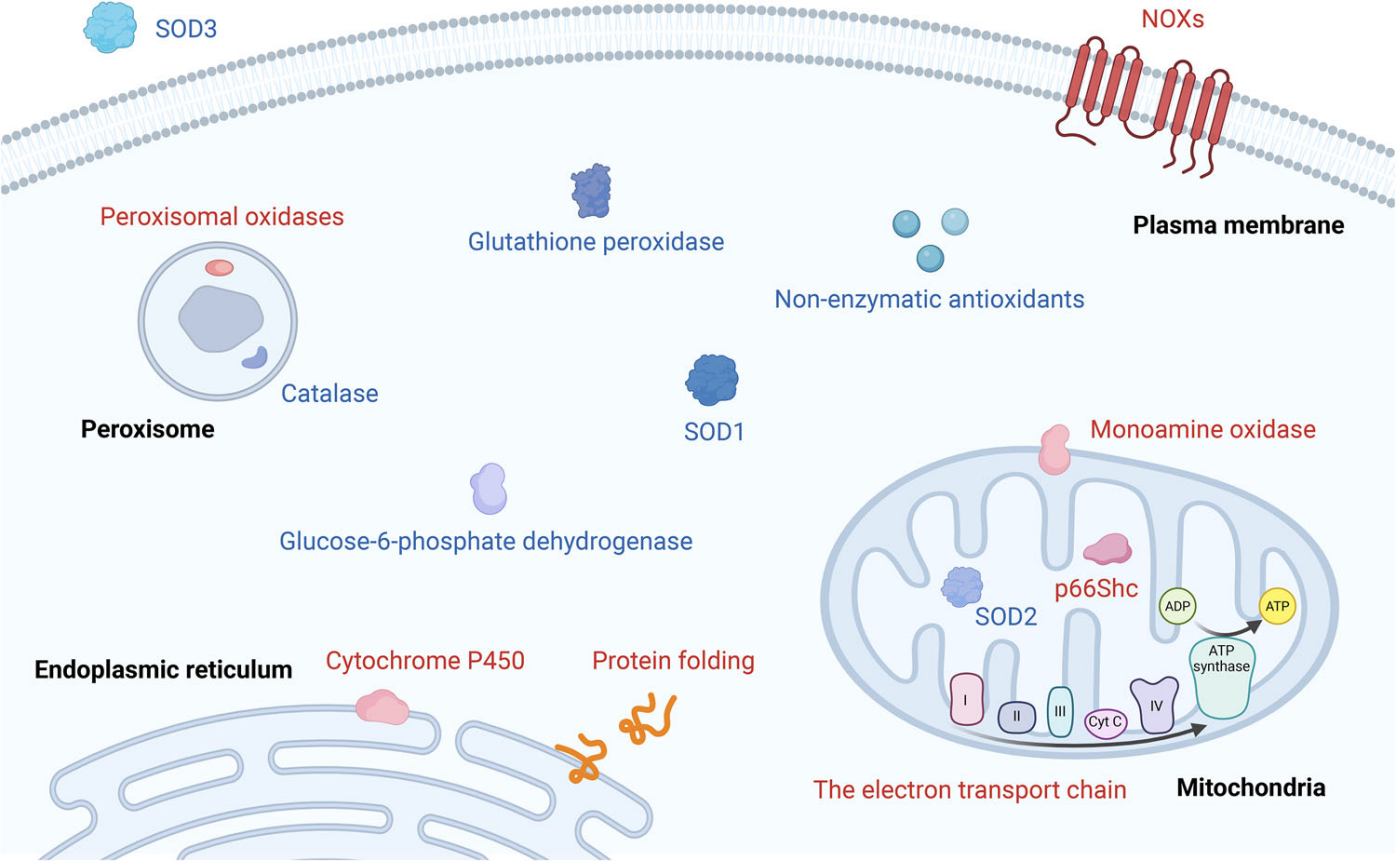
The production and elimination of reactive oxygen species (ROS). There are multiple intracellular sources of ROS, including the electron transport chain, monoamine oxidase, P66shc, the process of protein folding, cytochrome P450 enzymes, peroxisomal oxidases, and nicotinamide adenine dinucleotide phosphate oxidases (NOXs). Correspondingly, there are various non‐enzymatic antioxidants and antioxidant enzymes within the cells, including superoxide dismutase (SOD), catalase, glutathione peroxidase, and glucose‐6‐phosphate dehydrogenase. The sources of ROS are marked in red, while the antioxidant components are marked in blue.

There are multiple sites within cells that can generate and release ROS, such as mitochondria, endoplasmic reticulum, peroxisome, and plasma membrane [32]. Meanwhile, the multilayered antioxidant defense network composed of non‐enzymatic antioxidants and antioxidant enzymes within cells prevents the excessive accumulation of ROS [33, 34]. The precise regulation of ROS generation and elimination maintain the cellular redox state in a dynamic equilibrium (Figure 2).

### 2.1 The Intracellular Sources of ROS

The ROS production and relative contributions from different sources vary with cell types and physiological conditions. The presence of both ROS consumers and ROS producers in the same subcellular structure means that only a net rate of ROS can be detected, which is much lower than the absolute rate of ROS production. However, detection of the net rate can indicate whether a particular subcellular structure exports or imports ROS [35]. Mitochondria are normally regarded as the main source of ROS in mammalian cells, whereas other subcellular structures also produce abundant ROS. Therefore, exploring other sites of intracellular ROS production is conducive to the precise regulation of oxidative stress.

Mitochondria serve as the main oxygen consumers and the primary source of ROS within cells. The electron transport chain (ETC), a series of electron‐carrier proteins located in the mitochondrial inner membrane, is the main contributor to mitochondrial ROS [36]. ETC receives hydrogen ions from nicotinamide adenine dinucleotide (NADH) or flavin adenine dinucleotide (FADH_2_) and deliver electrons to oxygen, ultimately generating ROS [37]. This process mainly occurs in complexes I and III. In complex I, NADH is oxidized to produce electrons, which are then transferred to oxygen to generate O_2_•^−^ [38]. In complex III, oxygen interacts with semiquinone and produces large amounts of O_2_•^−^ [39]. Subsequently, O_2_•^−^ will be rapidly converted into H_2_O_2_ by manganese superoxide dismutase (MnSOD) within the mitochondrial matrix and then transmitted to the cytoplasm to participate in other biological reactions [40]. In addition to ETC, other ROS production sites exist in mitochondria including monoamine oxidase (MAO) and p66shc. MAO is localized to the outer mitochondrial membrane, where it catalyzes the oxidative deamination of aromatic amines such as catecholamines and generates H_2_O_2_ [41]. Physiologically, MAO plays a crucial role in the termination of neurotransmitter signal conduction in the nervous system and serves as a biomarker for neurodegenerative and psychiatric disorders [42]. Exacerbated MAO activity in pathological conditions can induce oxidative stress and cause harmful effects such as cardiovascular diseases [43]. p66Shc, a member of the Src homologous‐collagen homologue adaptor protein family, is an oxidoreductase that produces ROS in a mitochondria‐dependent manner. Specifically, p66Shc can generate ROS through the oxidation of cytochrome c by utilizing the reducing equivalents of the mitochondrial ETC, playing a crucial role in carcinogenesis and aging [44].

Endoplasmic reticulum is the largest intracellular organelle comprising a variety of tubules and sheet‐like structures spanning the cytoplasm. Physiologically, endoplasmic reticulum is responsible for several cellular functions including protein biosynthesis, folding, and transport, calcium storage and gated release, and lipid biosynthesis [45]. The formation of intramolecular disulfide bond is one of the most common post‐translational modifications occurring within the endoplasmic reticulum and is crucial for the production of correctly folded proteins. A highly oxidized environment is essential for this process, which is maintained by a series of enzymatic components especially endoplasmic reticulum oxidoreductase 1 (ERO1) and protein disulfide isomerase (PDI) [46]. ERO1 can utilize flavin adenine dinucleotide (FAD) as an electron acceptor to promote the formation of disulfide bond, which is then transferred to nascent protein substrates via PDI. Due to the reoxidation of the reduced FAD through reaction with oxygen, the ERO1 activity contributes to the generation of H_2_O_2_ as a by‐product [47]. In addition to the protein folding, enzymatic reactions within endoplasmic reticulum can also produce ROS. The cytochrome P450 (CYP) enzymes are a diverse group of heme monooxygenases that play a significant role in drug metabolism and lipid biosynthesis. In the presence of oxygen, CYP enzymes catalyze the hydroxylation of a hydrocarbon substrate with the assistance of electron donors. Nevertheless, ROS may leak in the intermediate steps of the reaction, known as “reaction decoupling,” which leads to elevated ROS levels and incomplete substrate oxidation [48]. There are various factors that determine the coupling efficiency of CYP reaction, such as the substrates and reaction microenvironment. For example, the stimulation of nasal epithelial cells with PM2.5 can increase ROS production through CYP1A1 [49].

Peroxisomes are single membrane‐bound organelles that contain a series of different enzymes. Owing to their high content of peroxisomal oxidases and antioxidant enzymes, peroxisomes play a central role in the dynamic balance of ROS generation and scavenging by catalyzing the dynamic spin of ROS, especially H_2_O_2_ [50]. Peroxisomes are multifunctional organelles that participate in the α‐oxidation of branched‐chain fatty acids, the β‐oxidation of very long chain fatty acids, the oxidation of D‐amino acids and polyamines, and the biosynthesis of phospholipids and bile acids [51]. As by‐products of these metabolic processes, a variety of oxidases present in peroxisomes produce ROS such as O_2_•^−^ and H_2_O_2._ Acyl‐CoA oxidases are the most abundant oxidases among almost all peroxisomes independent of the cell type. In addition, ROS‐producing oxidases include D‐amino acid oxidase, D‐aspartate oxidase, urate oxidase, and xanthine oxidase [52, 53]. Peroxisomes play an integral role in metabolic processes, thereby interacting and coordinating with other subcellular organelles, such as mitochondria, endoplasmic reticulum, lysosomes, and nucleus, which may also involve redox signaling [54, 55]. Notably, a recent study has shown that peroxisomes play a direct role in maintaining mitochondrial redox homeostasis through contact‐mediated ROS transfer. During this process, ACBD5 and PTPIP51 form the contact between peroxisomes and mitochondria. When the oxidative stress occurs in mitochondria, the percentage of contacts increase to transfer ROS from mitochondria to the lumen of peroxisomes [56]. This finding reveals the direct mechanism by which peroxisomes promote mitochondrial health and deepens our understanding of ROS transfer between subcellular organelles.

Nicotinamide adenine dinucleotide phosphate (NADPH) oxidases (NOXs) are a family of transmembrane enzymes proteins mainly located in the plasma membrane [57]. NOXs can generate electrons from NADPH and react with oxygen to produce O_2_•^−^ and H_2_O_2_, serving as the major source of nonmitochondrial ROS. The NOX family is comprised of seven members: NOX1 to NOX5, DUOX1, and DUOX2, which function either independently or in combination with one or more auxiliary subunits [58]. NOX1 to NOX3 require assembly of cytosolic regulatory subunits for activatoin, such as NOXA1, NOXO1, Tks 4/5, p40^phox^, p47^phox^, p67^phox^, and Rac. NOX4 has constitutive activity, and its catalytic activity can be enhanced by Poldip2 and Tks 4/5. In addition, the catalytic subunits of NOX1 to NOX4 combine with p22^phox^ to stabilize the complex. On the contrary, the activation of NOX5, DUOX1, and DUOX2 is calcium dependent and not dependent on the binding with p22^phox^ [59, 60]. The ROS generated by NOXs plays an integral role in various physiological processes, including innate immunity, redox signaling, and hormone production [61, 62].

### 2.2 The Multilayered Antioxidant Defense Network

Corresponding to the production of ROS, organisms have developed an antioxidant system to prevent the excessive accumulation of ROS and defend against oxidative stress. The antioxidant enzymes play a major role in combating the massive buildup of ROS due to their antioxidant activity. Meanwhile, the non‐enzymatic antioxidants are also important components of the antioxidant system. In reaction to oxidants and other electrophilic reagents, the antioxidant system is reinforced, thereby strengthening the capacity to resist oxidative stress [63, 64]. Hence, enhancing the antioxidant system has been the principal strategy of antioxidant treatment.

Superoxide dismutase (SOD) is the first line of antioxidant system and catalyzes the dismutation of O_2_•^−^ into H_2_O_2_, which is then decomposed by CAT and GPX. There are three distinct members of SOD family in mammals, including Cu/Zn‐SOD (SOD1), Mn‐SOD (SOD2), and extracellular SOD (EC‐SOD/SOD3), which exhibit structural diversity and all have free radical scavenging activities [65]. They have a wide range of subcellular localization: SOD1 is located in the cytoplasm, while SOD2 (Mn‐SOD) is found in the mitochondria, and SOD3 (EC‐SOD) is distributed in the extracellular matrix [66]. By virtue of this, the SOD family serves as an efficient and hierarchical antioxidant system. CAT, which is typically found in cellular peroxisomes, utilizes iron as a key component of the active site for the decomposition of H_2_O_2_ into oxygen and water. The three‐dimensional structure of CAT includes heme as a prosthetic group, which is essential for its catalytic efficiency [67]. GPX, which is primarily located in the cytoplasm and mitochondria, is an enzyme containing four units of selenium as cofactors. With the help of glutathione (GSH), GPX can reduce H_2_O_2_ or lipid peroxides [68]. In addition, glucose‐6‐phosphate dehydrogenase (G6PDH) is a housekeeping enzyme that takes part in the pentose phosphate pathway and catalyzes the conversion of NADP^+^ into NADPH, which is crucial for reducing oxidative stress within cells [69, 70].

Generally, the catalytic efficiency of antioxidant enzymes is much higher than that of non‐enzymatic antioxidants. However, the subcellular localization or lack of metal ions may limit the antioxidant effect of enzymes. In this case, the non‐enzymatic antioxidants take over the fight against ROS. The non‐enzymatic antioxidants mainly consist of small molecular antioxidants with a molecular weight of less than 1 kDa, such as GSH, coenzyme Q10, vitamin C, vitamin E, carotenoids, and phenolic compounds. They can be directly involved in scavenging ROS to protect organisms from oxidative damage and participate in several physiological processes (Table 1).

**TABLE 1:** The main non‐enzymatic antioxidants in vivo.

Antioxidant	General description	Mechanism of action	Synthesized in vivo	Reference
Glutathione	A tripeptide composed of glutamic acid, cysteine, and glycine	Glutathione provides reducing equivalents for glutathione peroxidases and directly eliminates reactive oxygen species (ROS)	Yes	[71]
Coenzyme Q10	An isoprenylated benzoquinone also known as ubiquinone	Coenzyme Q10 directly scavenges ROS and inhibits lipid peroxidation	Yes	[72]
Vitamin C	A six‐carbon lactone	Vitamin C directly scavenges ROS and act as a cofactor for the hydroxylases involved in collagen biosynthesis and the TET dioxygenases	No	[73]
Vitamin E	A group of eight monophenols α‐, β‐, γ‐, δ‐tocopherols and α‐, β‐, γ‐, δ‐tocotrienols	Vitamin E directly eliminates ROS and inhibits lipid peroxidation	No	[74]
Carotenoids	A class of natural pigments, including β‐carotene, lutein, astaxanthin, and lycopene	Carotenoids directly scavenge ROS and can be enzymically cleaved to vitamin A	No	[75]
Phenolic compounds	Phenolic compounds include monophenols, such as caffeic, and polyphenols, such as anthocyanins	Phenolic compounds directly eliminate ROS	No	[76]

**FIGURE 3:**
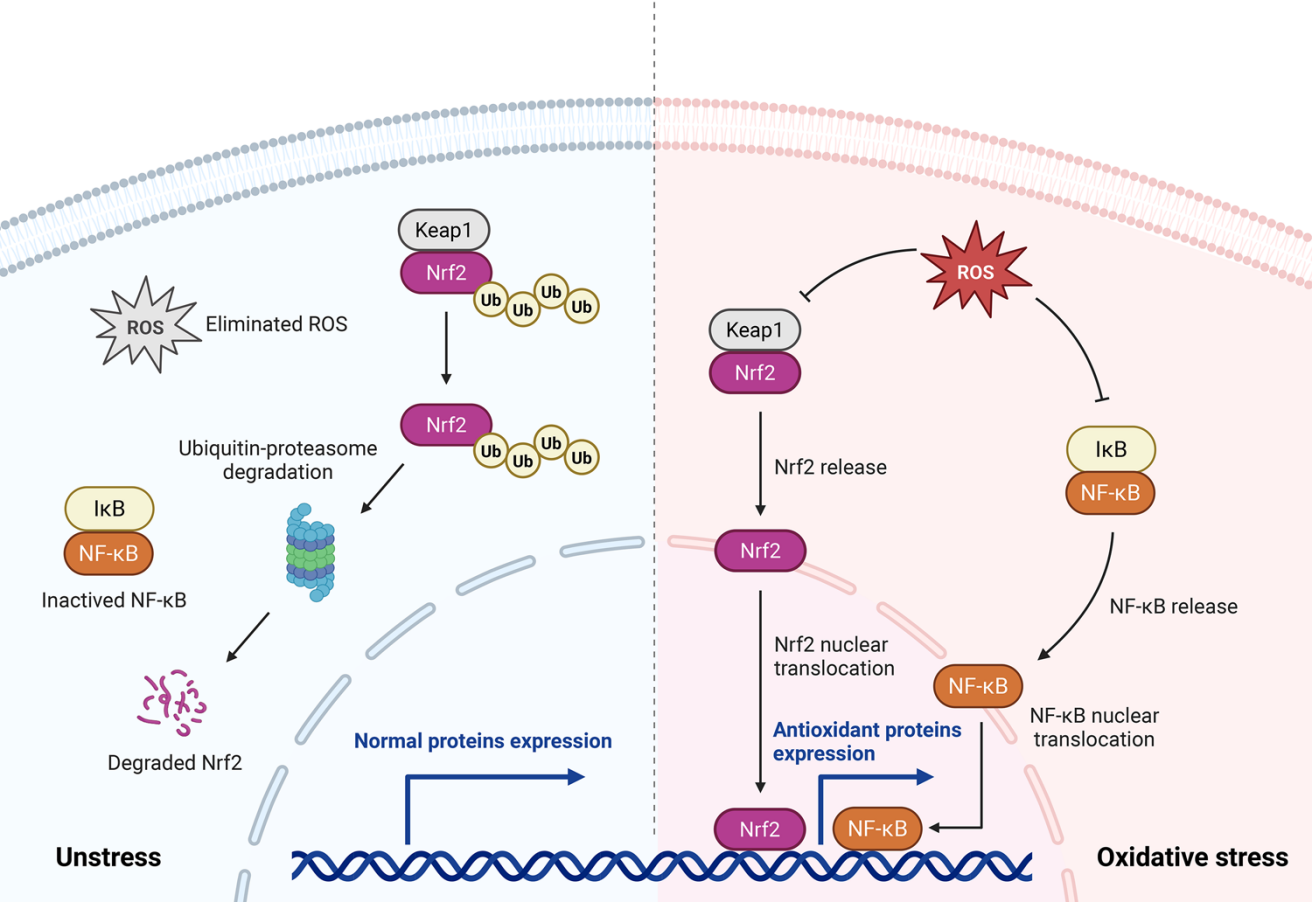
Oxidative stress activates the nuclear factor‐E2‐related factor 2/Kelch‐like ECH‐associated protein‐1(NRF2/KEAP1) and nuclear factor kappa‐B (NF‐κB) pathways. When ROS is eliminated in time, KEAP1 binds to NRF2 and promotes its degradation through the ubiquitin‐proteasome pathway, whereas under oxidative stress conditions, KEAP1 is modified and releases NRF2. The inhibitory subunit of the NF‐κB inhibitor (IκB) can also be modulated by ROS and activate NF‐κB pathway.

Molecular redox switches, mostly thiol‐based switches, sense ROS in a coordinated manner to control the oxidative state of cells. They take part in the regulation of the redox‐sensitive transcription factors, including NRF2/Kelch‐like ECH‐associated protein‐1 (NRF2/KEAP1) and nuclear factor kappa‐B (NF‐κB), which are regarded as the integral regulators of a wide range of biological processes (Figure 3).

NRF2 regulates the transcription of genes coding for antioxidant proteins and thereby protects against oxidative damage. Under normal unstressed conditions, KEAP1 binds to NRF2 and facilitates its degradation via the ubiquitin‐proteasome pathway, which is crucial for maintaining NRF2 at low levels [77]. However, under oxidative stress condition, specific cysteinyl residues of KEAP1 are modified, thereby preventing the binding of KEAP1 to NRF2. The stable NRF2 accumulate in the nucleus, where it binds to antioxidant response element (ARE) in the DNA and induces the expression of target genes [78]. The activity of NRF2 is highly dependent on several signaling pathways, including protein kinase RNA‐like endoplasmic reticulum kinase (PERK) pathway and mitogen‐activated protein kinase (MAPK) pathway, making it a complex and context‐dependent transcription factor [79, 80].

NF‐κB is a transcription factor that plays important roles in inflammation and immunity [81]. NF‐κB can be modulated by ROS and contribute to the release of the inhibitory subunit of the NF‐κB inhibitor (IκB). Then NF‐κB can translocate to the nucleus and induce the expression of target genes. As with NRF2, DNA‐binding subunits of NF‐κB contain redox‐sensitive cysteine residues, which are regulated by the nuclear thioredoxin‐1 [82, 83]. It is worth mentioning that there is crosstalk between NRF2 and NF‐κB pathways. NRF2 can inhibit the activation of NF‐κB by suppressing the degradation of IκB, whereas NF‐κB transcription inhibits NRF2 pathway by reducing ARE transcription [84].

## 3 Molecular Targets of Oxidative Stress

Although the physiological level of ROS is crucial for governing life processes through redox signaling, when ROS production exceeds the regulatory limits of the antioxidant system, excessive ROS can contribute to the oxidative damage of biomolecules, including nucleic acids, proteins, and lipids. Interestingly, oxidatively modified biomolecules can not only be counteracted by direct repair or restoration responses but also serve as signaling molecules to participate in redox regulation.

### 3.1 Genomic Instability and Epigenetic Regulation

Oxidative stress is one of the main factors that disrupts the integrity and stability of DNA. In the process of DNA replication or transcription, ROS can chemically induce DNA missense mutations, truncation mutations, and even break [85]. Among the DNA bases, guanine is most susceptible to oxidative damage. The main mutagenic lesion is 8‐oxoguanine (8‐oxo‐dG), which can base pair with adenine rather than cytosine and lead to transversion mutations once DNA replication is completed [86]. Base excision repair (BER) is the predominant repair system that removes oxidative DNA damage to bases. First, DNA repair‐related enzyme 8‐oxo‐dG glycosylase (OGG1) recognizes and removes the damaged base to form an apurinic (AP) site. Then, the sugar‐phosphate backbone is cleaved by AP endonuclease 1 (APE1) activity, and the resulting gap is filled with a single‐nucleotide match (short‐patch BER) or a few long matches (long‐patch BER) [87]. Redox signaling can also influence the repair of DNA damage by precisely regulating the oxidation of DNA repair‐related proteins. When the DNA double‐strand break occurs, the essential sensor ataxia‐telangiectasia mutated protein kinase (ATM) can be oxidized at the C‐terminal kinase domain (Cys2991), thereby being activated and facilitate the repair of DNA break [88, 89]. During the BER process, redox modifications can enhance the enzymatic activity of alkyl‐adenine DNA glycosylase (AAG) and promote DNA repair, but inhibit the activities of OGG1 [90, 91]. The different results reflect the diversity in specific cysteine site and biological functions of redox modifications. Similar to DNA, RNA can also suffer from oxidative damage, where 8‐oxo‐G in RNA compromises translational fidelity. Considering the longer sequences of noncoding RNA, the impact of 8‐oxo‐G modifications may be generally less significant. However, for shorter microRNAs, approximately 18–25 nucleotides in length, these modifications may have a more obvious effect [92, 93].

In addition to oxidative damage, ROS can regulate epigenetic modifications, which is a heritable change in gene function without alterations in DNA sequence. Interestingly, 8‐oxo‐dG is not only a marker of oxidative damage to nucleic acids, but also acts as an epigenetic mark that regulates gene expression [94]. When 8‐oxo‐dG is formed in potential quadruplex‐forming sequences (PQS) in promoter‐coding strands, its intermediate AP site unmasks the PQS and folds into a G‐quadruplex structure bound to APE1. The APE1 inefficiently cleaves but recruits various transcription factors to activate downstream genes, such as vascular endothelial growth factor (VEGF) and endonuclease III‐like protein 1 (NTHL1) [95]. Moreover, 8‐oxo‐dG near CpG islands can reduce the binding of DNA methyltransferases (DNMTs) and methyl‐CpG binding proteins, thus passively interfering with 5‐methylcytosine (5mC) [96, 97]. The 8‐oxo‐dG binding protein OGG1 can also recruit tet methylcytosine dioxygenase 1 (TET1) to the 8‐oxo‐dG lesion, which oxidizes adjacent 5mC to 5‐hydroxymethylcytosine (5hmC), thus regulating the transcription of downstream genes [98].

### 3.2 Proteostasis Imbalance

The proteostasis network is composed of the production, folding, and degradation of proteins, which is crucial for maintaining the function of the proteome. Oxidative stress can disrupt the proteostasis network, leading to the dysregulation of protein homeostasis and the accumulation of toxic protein aggregates [99]. Due to the sulfur and selenium‐containing amino acid side chains that are sensitive to oxidation, proteins are susceptible to oxidative modification by ROS. Interestingly, some redox sensitive proteins are involved in signaling events as reversible oxidative modifications, regulating adaptive responses to stimulation [100, 101]. For example, the thiol group of the cysteine side chain is subject to several oxidative modifications and can be oxidized to sulfenic, sulfinic, and sulfonic acids or converted to a disulfide, persulfide, or nitrosylated cysteine. Different oxidation states of cysteine exhibit distinct reactivity and act as redox switches to regulate protein functions [102]. However, excessive oxidative stress may lead to irreparable protein oxidation, such as backbone breakage, protein carbonylation, and covalent cross‐linking between tyrosine residues [103].

The endoplasmic reticulum is the location where oxidized proteins fold, typically forming disulfide bonds between the cysteine residues of proteins [104]. Many enzymes participate in this process, including the PDI family of dithiol‐disulfide oxidoreductases, ERO1, GPX7, and GPX8. Under the oxidative condition, activated ERO1 generates disulfide bonds by consuming oxygen in the presence of flavin cofactors, which are then transferred to the protein for folding through PDI. GPX7 and GPX8 can serve as oxidative stress sensors and utilize the H_2_O_2_ produced by ERO1 to facilitate protein folding [105]. Although oxidation is essential for protein oxidative folding, reduction is equally significant for suppressing the formation of incorrect disulfide bonds and maintaining the redox homeostasis of the ER. The enzymatic activity of ERO1 can be controlled through a negative feedback regulation mechanism to prevent ER peroxidation [106].

Misfolded peptides and oxidized damaged proteins are mainly degraded by the ubiquitin‐proteasome system and the autophagy‐lysosomal pathway [107]. The ubiquitin‐proteasome system itself is regulated by ROS. For instance, ubiquitinating enzymes can be inactivated by oxidative modifications, such as disulfide bond formation, S‐nitrosylation, and S‐glutathionylation. Moreover, the proteolytic activity of the proteasome requires catalysis by the intact sulfhydryl groups of Cys residues in the β subunit of the 20S core, which is susceptible to oxidative stress [26]. The autophagy‐lysosomal pathway serves as a secondary line of defense for degrading misfolded proteins. When highly oxidized proteins form cross‐linked aggregates that are difficult to be degraded by the ubiquitin‐proteasome system, they are redirected to degradation by the autophagy system [107]. Mild oxidative stress can upregulate the autophagy flux to eliminate aggregates and damaged organelles [108]. However, excessive oxidative stress may impair the autophagy‐lysosomal pathway and even lead to lysosomal membrane leakage, ultimately resulting in autophagic cell death [109, 110].

### 3.3 Lipid Peroxidation

Lipid peroxidation is an endogenous chain reaction composed of the oxidative degradation of lipids, which generates various oxidation products. Among them, the main primary products are lipid hydroperoxides, which are non‐radical intermediates derived from phospholipids, unsaturated fatty acids, glycolipids, cholesterol, and cholesterol esters [111, 112]. Both non‐enzymatic and enzymatic pathways participate in the formation of lipid hydroperoxides. ROS can attack lipids containing carbon‐carbon double bonds in cell membranes. Among them, polyunsaturated fatty acids (PUFAs), including arachidonic, linoleic, linolenic, and docosahexaenoic acids, are the most susceptible to oxidation [113]. As a specific class of ROS, excessive lipid peroxidation can disrupt plasma membrane integrity and further trigger multiple cell death modalities, including ferroptosis, apoptosis, necroptosis, pyroptosis, parthanatos, autophagy, cuproptosis, lysosomal‐dependent cell death, and NETosis [114]. Although these cell death patterns were generally recognized as independent process, numerous evidence has demonstrated that they are synergistically involved in the pathogenesis of disease [115]. The damage‐related molecular patterns released by cell death may transmit lipid peroxidation and trigger other cell death patterns. Oxidation products of lipids also play a significant role in oxidative signaling. For example, certain alkyl phospholipid oxidation products in oxidized low‐density lipoprotein are high‐affinity extracellular ligands for peroxisome proliferator‐activated receptor (PPAR) that induce PPAR‐responsive genes [116].

## 4 Oxidative Stress in the Pathogenesis of Disease

**FIGURE 4:**
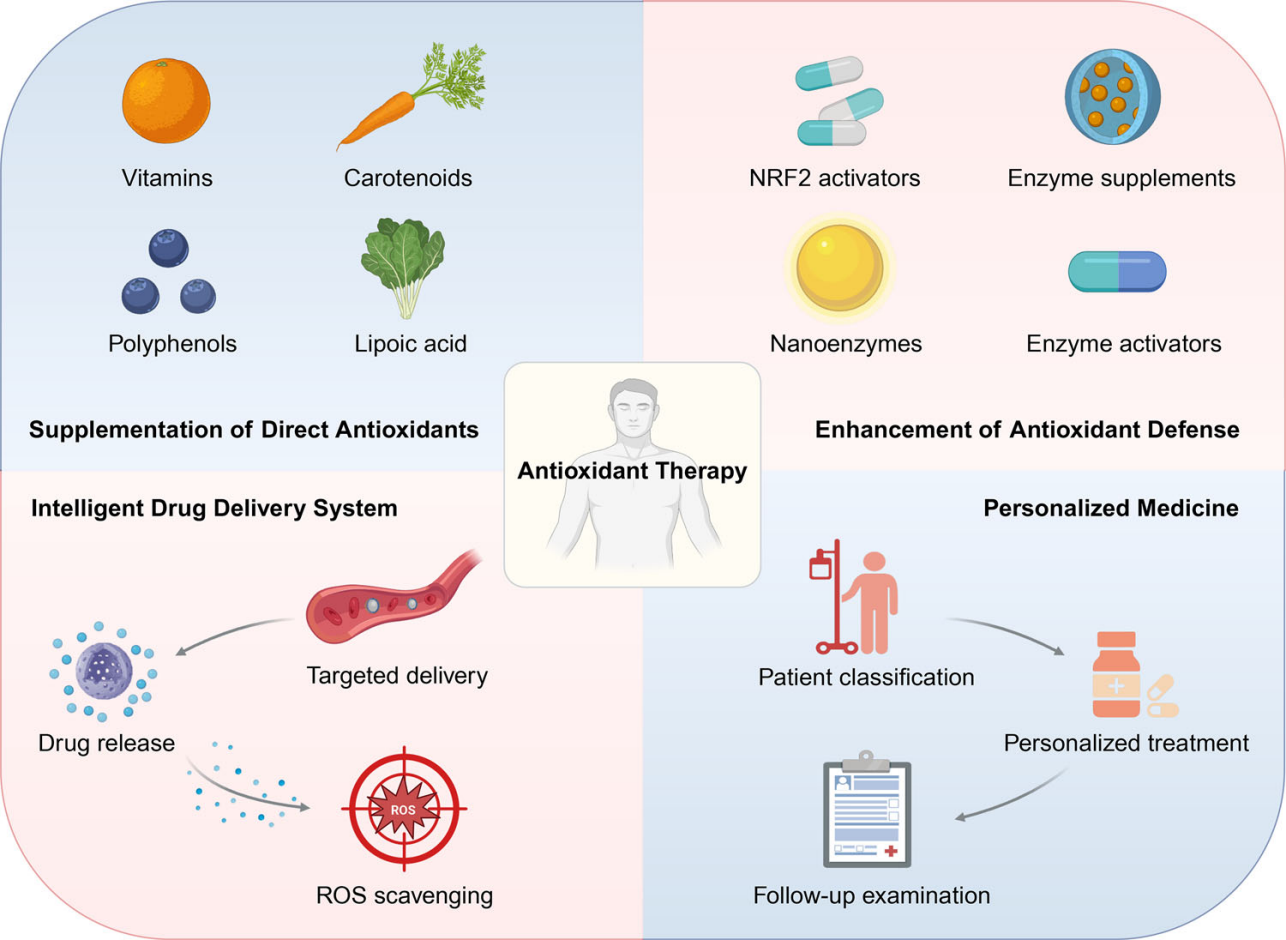
Therapeutic landscape targeting redox balance. Traditional antioxidant therapy, such as supplementation of direct antioxidants and enhancement of antioxidant defense, needs to confront challenges such as delivery difficulties and off‐target effects. Intelligent drug delivery system and personalized medicine are expected to overcome these limitations.

Oxidative stress participates in the pathogenesis of diverse inflammatory and metabolic diseases across multiple organs, including musculoskeletal diseases, cardiac diseases, dermatological diseases, cancer, obesity, diabetes, and hypertension (Figure 4). Oxidative stress can not only serve as the primary cause of pathology, but also act as the secondary contributor to disease progression. The research on antioxidants further emphasizes the pivotal role of oxidative stress within these specific systems. Antioxidant therapy has been proven to have satisfactory therapeutic effects in diseases where oxidative stress is the main pathological factor, but it may have adverse effects in the treatment of complex diseases. Hence, it is necessary to understand the mechanism by which oxidative stress promotes the occurrence and development of these diseases.

### 4.1 Oxidative Stress‐Induced Metabolic Syndrome

Oxidative stress serves as the central pathological nexus in metabolic syndrome, which is characterized by abdominal obesity, insulin resistance, inflammation, high blood pressure, and blood lipid disorders [117]. Metabolic syndrome is a series of interrelated metabolic disorders. The inflammatory environment caused by obesity can induce insulin resistance, and further lead to other metabolic comorbidities.

Oxidative stress operates as a central orchestrator in the pathophysiology of obesity, directly linking adipose tissue expansion to systemic metabolic dysfunction and chronic inflammation via ROS‐mediated mechanisms. In obese adipose tissue, excessive ROS production from NADPH oxidases and impaired ETCs induces mitochondrial DNA (mtDNA) damage, which escapes into the cytoplasm and circulation as damage‐associated molecular patterns (DAMPs), activating TLR9 and NLRP3 inflammasome pathways to trigger pro‐inflammatory cytokine cascades such as IL‐1β and IL‐18 [118, 119]. This oxidative environment promotes lipid peroxidation and protein modification, leading to insulin resistance and metabolic dysfunction. Recent studies highlight the novel “oxidative stress avoidance” mechanism mediated by lipopolysaccharide‐binding protein accumulation in lipid droplets, which serves as a hub connecting stress–oxidative stress–metabolic disorders–obesity [120]. Additionally, ROS‐induced ribosomal oxidation activates the ZAKα pathway and inhibits mitochondrial biogenesis and mitochondrial autophagy, thereby exacerbating ROS leakage and accelerating the dysfunction of adipose tissue [121].

Oxidative stress is closely associated with insulin resistance. Excessive ROS can activate MAPK, such as c‐Jun N‐terminal kinase (JNK), which phosphorylates specific serine/threonine sites on insulin receptor substrates (IRS) and inhibit their ability to interact with the insulin receptor [122]. In addition, oxidative stress contributes to insulin resistance in the periphery by disturbing glucose transport 4 (GLUT4), resulting in the maintenance of elevated blood glucose levels [123]. In pancreatic β‐cells, excessive production of ROS promotes β‐cell dysfunction and apoptosis, ultimately leading to insufficient insulin secretion [124]. Worse still, the prolonged hyperglycemia further causes metabolic disorders, including the aberration of the polyol pathway and hexosamine biosynthesis pathway, advanced glycation end (AGE) formation, and the activation of protein kinase C, all of which further augments ROS generation and form a vicious cycle [125, 126].

In the pathogenesis of cardiovascular diseases, excessive ROS production disrupts mitochondrial function, amplifies inflammatory responses, and induces significant cellular damage, such as myocardial fibrosis, cardiomyocyte apoptosis, and extracellular matrix degradation [127, 128]. The hyperglycemic milieu in diabetes and the activated renin‐angiotensin‐aldosterone system (RAAS) in hypertension converge to amplify ROS production, primarily through the synergistic activation of NOX isoforms and mitochondrial ETC dysfunction [129]. This ROS surge, particularly of superoxide anions, rapidly inactivates nitric oxide (NO) and uncouples nitric oxide synthase (eNOS), leading to impaired endothelium‐dependent relaxation and increased peripheral resistance [130]. In metabolic syndrome, multiple factors due to oxidative stress cooperate to promote the progression of hypertension, including inflammatory adipokines, activation of the sympathetic nervous system, abnormal gut microbiota, and retention of water and sodium [131]. In conclusion, oxidative stress plays a core role in the multi‐organ damage of metabolic syndrome and mediates various metabolic disorders.

### 4.2 Oxidative Stress‐Induced Cellular Senescence

Excessive ROS induces oxidative damage to various biomolecules, especially DNA, which is the quintessential signal for senescence induction [132]. DNA damage actives the DNA damage response (DDR), which is responsible for the activation of the p53‐p21 axis, thereby blocking cyclin‐dependent kinase 2 (CDK2) activity and triggering cell cycle arrest [133]. When the oxidative stress state persists and the damage progresses, senescent cells enter a senescent state marked by p16 upregulation, which consequently inhibits the kinases CDK4 and CDK6, thereby maintaining the stagnant state of the cell cycle [133, 134]. After exiting the cell cycle, cells enter into a hypersecretory state, secreting inflammatory cytokines, chemokines, and growth factors, which is termed senescence‐associated secretory phenotype (SASP) [135]. The DDR induced by oxidative stress also regulates SASP through the activation of NF‐κB signaling, which is one of the main transcription factors triggering SASP, such as IL‐6 and IL‐8 [136]. The SASP is the major mediator of the paracrine effects of senescent cells and drives secondary senescence.

The accumulation of senescent cells during aging leads to chronic inflammation, which contributes to organ and tissue decline. In neurodegenerative diseases such as Alzheimer's disease, the aging trajectory of the brain is closely linked to various cell types, including oligodendrocyte precursor cells, glia cells, neural stem cells, neurons, and endothelia [137]. Oxidative stress is regarded as a common feature of neurodegenerative diseases, which leads to telomere attrition, the excessive phosphorylation of tau protein, increased Aβ oligomers, and immunologic derangement, further mediating the senescence of these brain cells [138]. Therefore, antioxidant therapy is considered applicable to age‐related neurodegenerative diseases.

Cellular senescence caused by oxidative stress also plays a core role in the progression of musculoskeletal diseases. Due to the inefficient clearance of damaged cells in cartilage tissue, senescent chondrocytes significantly promote the progression of osteoarthritis through excessive secretion of SASP factors [139]. In response to SASP, macrophages are recruited into the articular space and secrete proinflammatory factors to activate synovial fibroblasts, which promote osteophyte formation and further exacerbating osteoarthritis [140]. Similarly, estrogen deficiency and aging cause accumulated senescent cells and increased SASP in the bone microenvironment. SASP facilitates the adipogenic differentiation, inhibits the osteogenic differentiation of bone marrow mesenchymal stem cells, and promotes the osteoclast formation, ultimately leading to osteoporosis [141].

### 4.3 Oxidative Stress‐Induced Cancer

Oxidative stress usually causes damage to nucleic acids, proteins, and lipids, which are associated with heightened risk of tumorigenesis [142]. Genomic instability induced by ROS is generally regarded as the main mutagenic force of tumor initiation, such as the mutation of the oncogene RAS and the tumor suppressor gene TP53. Meanwhile, these mutated genes can alter the metabolic signaling pathways such as hypoxia‐inducible factor 1 (HIF‐1) pathway, regulate glycolysis and mitochondrial function, and thus increase the production of ROS [143]. Worse still, the inactivation of TP53 can decrease the expression of antioxidant gene, including SOD2, GPX1, sestrin1 (SESN1), and SESN2 [144]. Therefore, cancer cells are often characterized by relatively high ROS levels.

The increased ROS level stimulates the proliferation and survival of cancer cells through various signaling pathways, including MAPK/extracellular‐regulated kinase (ERK) pathway, phosphoinositide‐3‐kinase (PI3K)/AKT pathway, and protein kinase D (PKD)/NF‐κB pathway [145]. In the tumor microenvironment, oxidative stress promotes the expression of VEGF and tumor angiogenesis, not only providing essential oxygen and nutrients for cancer cells but also involved in removing metabolic wastes [146]. Moreover, ROS can promote tumor metastasis. The epithelial‐to‐mesenchymal transition (EMT) has been recognized as a driving force in tumor metastasis, which is characterized by the loss of cellular adhesion and apical‐basal polarity [147]. ROS participates in this process by reducing cell–cell junctions, promoting cytoskeletal remodeling, and facilitating extracellular matrix degradation [148].

It is worth mentioning that ROS plays a dual role in cancer cells. Although ROS promotes the occurrence and progression of cancer, when it exceeds the compensation threshold, excessive ROS can induce various forms of cancer cell death, including apoptosis, necroptosis, ferroptosis, and pyroptosis [149]. ROS stimulates the extrinsic apoptotic pathway by promoting the ubiquitin degradation of the cellular FADD‐like IL‐1β‐converting enzyme‐inhibitory protein (c‐FLIP), which inhibits the formation of death‐inducing signaling complex (DISC) via competitively combining with FADD [150]. Furthermore, ROS mediates the intrinsic apoptosis by promoting the release of pro‐apoptotic factor cytochrome‐c from mitochondria to form apoptosome with apoptotic protease activating factor 1 (APAF‐1) and caspase‐9 [151]. Necroptosis is a caspase‐independent regulated form of cell death. ROS and necroptosis can form a necroptotic loop: ROS stabilizes receptor interacting protein kinase 3 (RIP3) to promote the necrosome formation, while RIP3 enhances ROS generation through metabolic signaling [152]. In addition, ferroptosis is a ROS‐driven and iron‐dependent regulated cell death. The oxidative stress induced by excessive intracellular iron levels and insufficient GSH contributes to the lethal accumulation of lipid peroxides, ultimately resulting in ferroptosis of cancer cells [153]. Pyroptosis is characterized by gasdermin (GSDM)‐mediated pore formation in the plasma membrane and pro‐inflammatory cytokines release. Overproduction of ROS can activate NLR family pyrin domain containing 3 (NLRP3), leading to the formation of inflammasome, maturation of caspase‐1, and activation of GSDMD, which creates pore structures to mediate inflammation [154]. Pyroptosis can enhance antitumor immune responses through pro‐inflammatory cytokines such as IL‐1β and IL‐18, whereas chronic inflammation induced by pyroptosis may promote tumor progression [155].

## 5 Therapeutic Targeting of the Redox Balance: Strategies and Challenges

Supplementation of direct antioxidants and enhancement of endogenous antioxidant defense network provide important strategies for the treatment of oxidative stress‐related diseases. Nevertheless, clinical trials that attempt to delay the onset or progression of such diseases are mostly negative. There are still some challenges in the process from basic research to clinical transformation of antioxidant therapy. Technological innovations represented by the intelligent drug delivery system and personalized medicine have also brought new opportunities. By adopting these new technologies, it is expected to overcome the limitations of traditional antioxidant therapy (Figure 4).

### 5.1 Supplementation of Direct Antioxidants

There are various antioxidants with the ability to directly eliminate ROS, such as vitamins, carotenoids, polyphenols, and lipoic acid, which are widely present in food and are often taken as dietary supplements [118, 119, 120, 121]. Nevertheless, since the role of ROS in disease is complex, treatments using direct antioxidants run the risk of oversimplification. The physiological effects of ROS need to be fully considered in the use of antioxidants. As mentioned earlier, antioxidant therapy for treating neurodegenerative and cardiovascular diseases holds potential. However, ROS plays significant physiological roles in the nervous system and cardiac function, which could be interfered with [156, 157]. In terms of the immune system, ROS at the physiological level can serve as signal molecules that trigger protective stress responses, and antioxidants can be counterproductive by inhibiting the natural adaptive response. Therefore, accurately identifying the key regulatory proteins and redox modification sites involved in the progression of diseases has become a research hotspot in this field [158, 159]. In addition, although the connection between the generation of free radicals and the pathogenesis of various diseases has been established, free radicals are often the result of developed diseases rather than the cause of their onset. Mechanisms accounting for this include recruitment of ROS‐generating phagocytes to the damaged tissues and activation of pro‐oxidant enzymes [160]. Even if antioxidant defense is significantly increased and oxidative stress markers are reduced, antioxidant treatment may have no significant impact on disease progression [161]. Worse still, the supplementation of antioxidants can promote the development of diseases in specific pathological conditions. For example, oxidative stress caused by smoking causes lung tissue damage, whereas supplementation with beta‐carotene as an antioxidant therapy may increase lung cancer incidence [162].

Another challenge in the antioxidant therapy is the effective delivery of antioxidants. The pharmacokinetics and target affinity of different antioxidants vary greatly. The reason why some clinical trials have not achieved the expected results may be that the antioxidants have not reached the expected action site and maintained an effective drug concentration [163]. To increase the effective concentration of antioxidants, some trials used high doses of antioxidants, which in turn promote oxidation. For instance, intravenous infusion of high dose vitamin C can produce cytotoxic quantities of H_2_O_2_ in the presence of transition metal ions [164]. Moreover, even if antioxidants reach the correct position, they may not reduce the specific molecular oxidative damage that causes diseases. For example, vitamin E can inhibit lipid peroxidation, but its effect on oxidative damage of proteins in Alzheimer's disease is limited [165, 166]. In other words, the complex interaction between ROS and antioxidants in oxidative stress‐related diseases is the most easily overlooked factor in clinical trials and leads to disappointing results.

### 5.2 Enhancement of Endogenous Antioxidant Defense Network

Considering the clearance rate of ROS by endogenous antioxidant defense network is often much higher than that of exogenous antioxidants, increasing endogenous antioxidant levels is a promising antioxidant therapy [167]. The NRF2 pathway is regarded as the most important system controlling antioxidant defense in vivo, which can resist oxidative stress damage. The NRF2 activators are mainly divided into two classes: cysteine‐reactive activators and PPI disrupting activators. The cysteine‐reactive activators target specific cysteine residues on KEAP1, resulting in the inhibition of the KEAP1‐E3 ubiquitin ligase and stabilization of NRF2. The PPI disrupting activators specifically target the KEAP1‐NRF2 interaction by binding to the KELCH domain of KEAP1, thereby disrupting the interaction with the ETGE motif of NRF2 [168]. Exercise is widely recognized to improve human health at least partly due to the activation of the NRF2 pathway [169, 170]. Several NRF2 activators are currently undergoing clinical trials, including dimethyl fumarate, sulforaphane, bardoxolone‐methyl, and omaveloxolone, whereas the pharmacokinetics issues and off‐target effects remain challenges [171, 172, 173, 174]. The NRF2 activators are promising in treatment of acute injuries such as ischemia‐reperfusion by protecting cells from oxidative damages [175]. Nevertheless, in chronic diseases where the NRF2 pathway is continuously activated or the response is impaired, their therapeutic effects may be limited.

It is worth noting that there may be a paradox in the NRF2‐based antioxidant therapy, such as in the cases of aging and cancer. During the aging process, the expression of NRF2‐dependent antioxidant enzymes decreases, thereby leading to oxidative stress and related diseases [176]. However, activating NRF2 may increase the risk of cancer in the elderly, as NRF2 activation protects cancer cells from oxidative damage, which contributes to the progression of cancer [177, 178]. The dual effects of NRF2 makes it a complex and interesting therapeutic target. NRF2 inhibitors such as brusatol have been explored for cancer treatment, and NRF2 activators show promise in preventing cancer or protecting cells from acute oxidative damages such as radiation during cancer treatment [179].

Additionally, exogenous supplementation of antioxidant enzymes or enhancing their ability to decompose ROS can resist oxidative stress. Natural antioxidant enzymes are usually susceptible to changes in temperature, pH, and chemical environment, leading to a decrease in their activity or complete inactivation [180]. Hence, it is necessary to use carriers such as metal‐organic frameworks (MOFs) to enhance the stability of antioxidant enzymes and promote their targeted release [181]. Nanoenzymes that can simulate the catalytic process of natural antioxidant enzymes has also been a research hotspot. For instance, cerium oxide and Prussian blue exhibit antioxidant properties due to their multi‐enzyme activities similar to SOD and CAT, and simultaneously possess properties unique to nanomaterials such as high catalytic activity, good stability, and low cost [182]. Furthermore, a recent study reported a small molecule, KDS12025, which can increase the activity of hemoglobin pseudoperoxidase by 100‐fold even at low hemoglobin levels. KDS12025 restores the redox equilibrium and prevents neurodegeneration, holding a great promise in oxidative stress‐related diseases [183].

### 5.3 Current Clinical Trials: Lessons From Failures

To date, numerous clinical trials have investigated the efficacy of antioxidants in oxidative stress‐related diseases, although many of them have reported failure results. General systemic antioxidant therapy often fails to achieve the desired therapeutic effect, which emphasizes the importance of personalized and precision medicine. The relevant clinical trials reported in the past 5 years are summarized below (Table 2).

**TABLE 2:** Clinical trials of antioxidant therapy over the past 5 years.

Registration number	Participants	Interventions	Results	Reference
NCT05829382	30 overweight healthy elderly	Oral 150 mg polyphenol capsules once daily for a week	Polyphenol supplementation did not change the blood levels of oxidative stress and inflammatory markers except for 4‐hydroxynonenal (4‐HNE)	[185]
NCT03161028	115 patients with progressive multiple sclerosis	Oral 1200 mg lipoic acid once daily for 24 months	Lipoic acid supplementation did not alleviate the decline in walking speed	[188]
NCT02832648	120 postmenopausal women with osteopenia	Oral 200 or 50 µg selenite once daily for 6 months	Selenium supplementation at these doses did not affect musculoskeletal health	[191]
ChiCTR‐IIR‐17012604	968 patients with chronic obstructive pulmonary disease (COPD)	Oral 600 mg N‐acetylcysteine (NAC) twice daily for 2 years	NAC treatment failed to improve lung function or reduce the annual rate of total exacerbations	[194]
ChiCTR1900021269	105 patients with poor ovarian response	Pretreatment with 4 IU growth hormone daily on day 2 of the previous menstrual cycle before in vitro fertilization until the trigger day	Pretreatment with growth hormone significantly increased the rates of higher quality embryo formation and clinical pregnancy	[197]
NCT04788745	21 patients with amyotrophic lateral sclerosis	Oral 35 mg trimetazidine twice daily for 12 weeks	Trimetazidine reduced the oxidative stress markers such as malondialdehyde and 8‐oxo‐dG and the resting energy expenditure	[199]

Polyphenols are a large group of natural antioxidant compounds commonly found in fruits and vegetables [184]. Chong et al. produced polyphenol capsules containing 150 mg phytonutrients including quercetin, catechins, phloretin, ellagic acid, and anthocyanins, which aimed to replicate elements from the mediterranean diet. After a week of continuous capsule administration in healthy overweight older adults, there were no significant changes in blood levels of oxidative stress and inflammatory markers other than 4‐hydroxynonenal (4‐HNE) [185]. The reason for the weak effect may be that the intervention duration is too short to produce any significant change in the biological mechanism.

As a cofactor for the mitochondrial enzymes including pyruvate dehydrogenase and alpha‐ketoglutarate dehydrogenase, lipoic acid can exert antioxidant effects by directly eliminating ROS, chelating with metal ions, and enhancing endogenous antioxidant capacity [186, 187]. Spain et al. attempted to use lipoic acid to treat progressive multiple sclerosis. The administration of 1200 mg oral lipoic acid daily for 24 months seems to protect the whole‐brain volume of patients, whereas the decline in walking speed was not alleviated [188]. Worse still, the lipoic acid group showed more proteinuria. This highlights the potential risks of over‐the‐counter dietary supplements, which may not have undergone systematic safety assessments.

Selenium is an essential trace element and a component of selenium compounds such as GPX. It can act as an enzymatic active center in redox reactions, thereby inhibiting oxidative stress [189, 190]. Walsh et al. evaluated the effect of selenium supplementation on the musculoskeletal health of postmenopausal women with osteopenia. The results demonstrated that 200 µg or 50 µg oral selenite for 6 months increased serum selenium, but did not affect urine N‐terminal cross‐linking telopeptide of type I collagen to creatinine ratio or bone mineral density [191]. Despite the epidemiological, observational and preclinical data all indicating the benefits of selenium for musculoskeletal health, this trial still achieved disappointing results, which may suggest the limitations of the effect of a single antioxidant.

The cysteine prodrug N‐acetylcysteine (NAC) is a widely used antioxidant, serving as a reducing agent of disulfide bonds, a scavenger of ROS, and a precursor for GSH biosynthesis [192, 193]. Zhou et al. treated patients with mild‐to‐moderate chronic obstructive pulmonary disease (COPD) with 600 mg oral NAC twice daily for 2 years. However, the long‐term and high‐dose treatment with NAC failed to improve lung function or reduce the annual rate of total exacerbations [194]. Interestingly, a greater benefit from NAC treatment was observed in former smokers, patients with COPD GOLD stage 2, and those with COPD exacerbation in the previous year, suggesting the impact of individual differences in patients on antioxidant treatment.

Meanwhile, some clinical trials of antioxidant therapy exhibited satisfactory results. Growth hormone has been reported to reduce oxidative stress in cardiac muscle and skeletal muscle [195, 196]. Gone et al. investigated the therapeutic effect of growth hormone on in vitro fertilization and embryo transfer (IVF‐ET) of poor ovarian responders, who received pretreatment with growth hormone 4 IU daily on day 2 of the previous menstrual cycle before in vitro fertilization until the trigger day. The results indicated that pretreatment with growth hormone significantly increased the rates of higher quality embryo formation and clinical pregnancy [197]. It should be noted that although reduced oxidative stress was detected in patients who received pretreatment with growth hormone, the therapeutic effect may be partly due to the biological effects of growth hormone.

Trimetazidine is a small molecule piperazine derivative that reduces fatty acid β‐oxidation, thereby inhibiting oxidative stress caused by lipid oxidative degradation [198]. Eijk et al. explored the effect of trimetazidine on oxidative stress markers and energy expenditure in amyotrophic lateral sclerosis. After treatment of 35 mg oral trimetazidine twice daily for 12 weeks, the oxidative stress markers, including malondialdehyde and 8‐oxo‐dG, and the resting energy expenditure were reduced in patients with amyotrophic lateral sclerosis, which may affect the disease progression of amyotrophic lateral sclerosis [199]. This trial presented positive results, whereas the larger long‐term randomized controlled trials are needed to confirm the therapeutic effect of trimetazidine on amyotrophic lateral sclerosis.

### 5.4 Future Perspectives: Intelligent Drug Delivery System and Personalized Medicine

Oxidative stress signals are essential for normal physiological signal transduction and exhibit spatiotemporal heterogeneity in disease progression. Therefore, antioxidant therapy requires precisely targeted delivery of drugs to the sites of oxidative stress damage. The progress of nanomedicine delivery systems has brought new opportunities to overcome these challenges. First, nanocarrier can play a protective role to enhance the in vivo stability and bioavailability of the loaded drug. For example, antioxidant polyphenols and metal ions can self‐assemble into nanoparticles through coordination, thereby improving water solubility and pharmacokinetic characteristics [200]. Moreover, through the modification of tissue targeting groups and microenvironment responsive groups, nanomedicine can respond to the high ROS levels at the oxidative damage site for precise drug delivery, enhancing the therapeutic effect and reducing the side effects [201, 202]. Furthermore, advanced nanomedicine attempts to integrate functions such as anti‐inflammation, immune regulation, and photothermal therapy with antioxidation. Considering that oxidative stress generally interacts with inflammation to form a complex pathological microenvironment, multifunctional nanomedicine may achieve a synergistic effect and obtain optimal therapeutic outcomes [203, 204].

The levels of oxidative damage and antioxidants vary widely between individuals, as do responses to oxidative stress damage and supplementation of antioxidants [205, 206]. This highlights the necessity of classifying patients based on the progression of oxidative stress diseases and developing targeted antioxidant drugs, which is known as personalized and precision medicine [207]. The precise regulation of redox balance is undoubtedly difficult, so the application of antioxidant therapy should be cautious before a systematic and comprehensive clinical evaluation, and the treatment plan should be adjusted based on follow‐up examinations. Future research should focus on elucidating the specific mechanisms of oxidative stress rather than the general “antioxidant therapy,” including the specific molecules regulated by oxidative stress in particular diseases, the subspatial distribution of ROS and antioxidants, as well as the differences in oxidative stress states between animal models and humans.

## 6 Conclusion

The production and elimination of ROS are in a dynamic equilibrium state, thereby maintaining the redox homeostasis. When the balance between oxidation and antioxidation in the body is disrupted, oxidative stress occurs with the accumulation of ROS and causes damage to biomolecules. As oxidative stress plays a crucial role in the occurrence and progression of diseases, the development of effective antioxidant therapy is a significant goal. The development of antioxidants that can reduce oxidative damage to specific biomolecules without interfering with the physiological functions of ROS has become a research focus. The development of novel technologies is conducive to overcoming the limitations of traditional antioxidant treatments. By precisely regulating redox signaling, it is expected to achieve precise treatment of various diseases. To better transform basic research achievements into clinical applications, further in‐depth research is still needed in this field.

## Author Contributions

Yi Qin and Chen Qian conceived and drafted the manuscript. Yi Qin made the figures and tables. Wenhao Li, Qihan Wang, Qifeng Shen, Zheqing Chen, Wei Zhang, and Wenming Li edited and revised the manuscript. Dechun Geng, Zhanjun Yan, and Gaoran Ge supervised and revised the manuscript. All the authors have read and approved the final manuscript.

## Funding

This work was supported by the National Natural Science Foundation of China (82472525, 82272567 and 82502941), Suzhou Technology R&D Program (Healthcare Innovation) Project (SYW2025005), Suzhou Outstanding Youth Pilot Program (SSD2025027), Science and Education Project of Suzhou (QNXM2024001), BoXi Talent Cultivation Program (BXQN2024006), Suzhou Medical College‐Qilu Medical Research Program of Soochow University (24QL200203), the Project of MOE Key Laboratory of Geriatric Diseases and Immunology (KJS2502), the Priority Academic Program Development of Jiangsu Higher Education Institutions (PAPD), Postgraduate Research & Practice Innovation Program of Jiangsu Province (SJCX25_1798), Suzhou Municipal Medical and Health Science and Technology Innovation Project (SKY2021023), Ke Jiao Xing Wei Plan of Wujiang District (WWK202005).

## Ethics Statement

The authors have nothing to report.

## Conflicts of Interest

The authors declare no conflicts of interest.

## Data Availability

Data availability is not applicable to this review as no new data were created or analyzed in this study.
